# Association of Pre-PCI Blood Pressure and No-Reflow in Patients with Acute ST-Elevation Coronary Infarction

**DOI:** 10.5334/gh.1309

**Published:** 2024-03-04

**Authors:** Xiaobo Li, Chen Yu, Li Lei, Xuewei Liu, Yejia Chen, Yutian Wang, ShiFeng Qiu, Jiancheng Xiu

**Affiliations:** 1 Department of Cardiology, Nanfang Hospital, Southern Medical University, Guangzhou, Guangdong, China; 2 Department of Cardiology, Xiangdong Hospital, Hunan Normal University, Liling, Hunan, China; 3 The Tenth Affiliated Hospital of Southern Medical University (Dongguan People’s Hospital), Southern Medical University, Dongguan, Guangdong, China

**Keywords:** **STEMI** (ST-elevation acute coronary infarction), **no**-reflow, **SBP** (Systolic blood pressure), **DBP** (Diastolic blood pressure)

## Abstract

**Background:**

Previous studies have established blood pressure (BP) as a pivotal factor influencing no-reflow following primary percutaneous coronary intervention (PCI) in patients with ST-elevation acute coronary infarction (STEMI). However, no relevant study has been conducted to investigate the optimal range of BP associated with the lowest risk of no-reflow among STEMI patients so far. Therefore, our objective was to evaluate the association between pre-PCI BP and the occurrence of no-reflow in patients with STEMI.

**Method:**

We included 1025 STEMI patients undergoing primary PCI. The BP pre-PCI was categorized into 20-mmHg increments. Logistic models were employed to assess the association of no-reflow with systolic blood pressure (SBP) or diastolic blood pressure (DBP). Three sensitivity analyses were conducted to further confirm the robustness of the association between blood pressure and no-reflow.

**Results:**

SBP or DBP exhibited a U-shaped curve association with no-reflow. No-reflow was higher in patients with lower SBP (<100 mmHg) (adjusted hazard ratio (OR) 3.64, 95% confidence interval (CI) 1.84,7.21; p < 0.001) and lower DBP (<60 mmHg) (OR 3.28, 95% CI 1.63,6.49; p < 0.001) [reference: 120 ≤SBP <140; 80 ≤DBP <100 mmHg], respectively. Furthermore, no-reflow was higher in patients with higher SBP (≥160 mmHg) (OR 2.07, 95% CI 1.27,3.36; p = 0.003) and DBP (≥100 mmHg) (OR 3.36, 95% CI 2.07,5.46; p < 0.001), respectively. The results of sensitivity analyses were consistent with the above findings.

**Conclusion:**

Maintaining a pre-PCI SBP within the range of 120 to 140 mmHg and a DBP within the range of 80 to 100 mmHg may be confer benefits to patients with STEMI in no-reflow.

## Introduction

Cardiovascular disease (CVD), particularly ST-segment elevation myocardial infarction (STEMI), stands as the leading cause of death in industrialized nations, with its incidence and mortality increasing at an unprecedented rate in some low- and middle-income countries [1]. Primary percutaneous coronary intervention (pPCI) represents the preferred reperfusion strategy in patients with STEMI according to current international guidelines from the European Society of Cardiology (ESC) [2]. Further, the establishment of chest pain centers (CPCs) has optimized emergency PCI processes, enabling earlier treatment and reducing mortality and complications among patients [3]. However, despite these advancements, some patients still experience unsatisfactory clinical outcomes, with a high mortality rate even after timely pPCI.

Studies have shown that 10–30% of patients with acute myocardial infarction (AMI) encounter the no-reflow phenomenon after successful pPCI [4]. The no-reflow phenomenon, defined as the lack of intramyocardial reperfusion following successful coronary recanalization in STEMI patients [5], amplifies the extent of myocardial ischemic necrosis and elevates the risk for adverse cardiovascular events such as in-hospital death, malignant arrhythmia, cardiogenic shock, and heart failure [6,7,8,9,10]. Consequently, this phenomenon offsets the cardiovascular benefits provided by CPCs in terms of timely vascular recanalization. Distal atherothrombotic embolization, ischemic damage, reperfusion injury, and individual susceptibility to microvascular damage are considered the primary pathophysiological mechanisms contributing to this phenomenon [11]. Despite extensive research on therapies targeting various known pathophysiological mechanisms underlying coronary no-reflow, no therapy has proven effective enough to prevent or reverse no-reflow while sustaining clinical benefit for STEMI patients undergoing PCI for reperfusion. Effectively avoiding or reducing the occurrence of no-reflow has become a focal point for improving the prognosis of patients with STEMI.

In the process of emergency PCI in patients with STEMI, monitoring blood pressure is crucial as it serves as a lifeline and a key indicator throughout the procedure [12]. Blood pressure also plays a significant role in coronary perfusion and the occurrence of the no-reflow phenomenon [13]. Ischemic heart disease severely impairs endothelium-dependent vasodilation in the coronary vascular system [14,15], and excessive low blood pressure can reduce coronary perfusion pressure, leading to no-reflow [16]. A study has identified admission systolic blood pressure (SBP) below 100 mmHg as an independent predictor for no-reflow during emergency PCI for STEMI patients [17]. Conversely, excessive elevation of blood pressure may increase microvascular resistance, attenuate myocardial blood flow response to afterload, and result in increased cardiac work [18,19]. Furthermore, elevated reperfusion pressure can exacerbate microembolization of red-cell, neutrophil, and platelet aggregates, contributing further to no-reflow by pushing these aggregates distally into smaller vessels [20]. Therefore, increasing blood pressure may not necessarily result in a corresponding increase in coronary flow; instead, it could worsen the occurrence of no-reflow. However, there are currently no relevant studies investigating the optimal range of blood pressure associated with the lowest risk of no-reflow among STEMI patients. In this retrospective cohort study, we evaluated the association between pre-PCI blood pressure (systolic and diastolic blood pressure) and the occurrence of no-reflow in patients with STEMI undergoing emergency PCI according to the chest pain center process, while exploring an optimal range that minimizes this risk.

## Methods

### Study design and participants

This cohort study enrolled 1050 STEMI patients who underwent primary PCI at Xiangdong Hospital, Hunan Normal University between December 30, 2016 and May 13, 2023. The following individuals were excluded: patients who died during PCI, patients who underwent thrombolytic therapy within 12 hours of onset, patients with severe mechanical complications, and those for whom blood pressure was not recorded before PCI. After excluding specific patients, 1025 (782 men and 243 women) were ultimately included in the study. The detailed screen process is depicted in Supplementary Figure 1.

According to the Thrombolysis in Myocardial Infarction (TIMI) classification of coronary blood flow after percutaneous transluminal coronary angioplasty or stent placement, the 1025 patients were categorized into two groups: reflow [TIMI = 3] and no-reflow [TIMI <3] (Supplementary Figure 1).

The research protocol adheres to the ethical guidelines of the 1975 Declaration of Helsinki. Approval for this study was obtained from the Ethics Committee of Xiangdong Hospital, Hunan Normal University (No.2020012). This clinical study follows a retrospective research design, collecting relevant patient indicators for statistical analysis, without additional intervention measures for the subjects. Risks to the subjects are limited to those associated with routine treatment, unrelated to this study. The sole risk involves the protection of privacy; therefore, all research materials had all identifiable patient information removed. Formal consent is not required for this type of study.

### Data Collection

General clinical data, data during the PCI procedure, and rapidly accessible pre-operative laboratory data of all patients were collected. The data were recorded on the chest pain form, electronic medical record system, and the Chinese chest pain center data submission website.

General clinical data included the following variables: gender, age, transfer from another hospital, drug treatment by another hospital, the number of ST elevation leads greater than 3, hypertension, hyperlipemia, diabetes, smoking history, Killip classification on admission, time from onset of chest pain to the PCI procedure, cardiac arrest occurrence before PCI, oral blood pressure medication before PCI, heart rate, systolic blood pressure, and diastolic blood pressure on admission.

Laboratory data included high-sensitivity troponin, hemoglobin, red blood cell count, white blood cell count, neutrophil count, platelet count, lymphocyte count, glucose, mean platelet volume, sodium (Na), potassium (K), and fibrinogen.

Data during PCI procedure encompassed heart failure events and malignant arrhythmias during PCI, Thrombus-shadow during coronary angiogram, lesion-vessel identification, the number of stents used, intra-aortic balloon pump (IABP) utilization, and intravenous blood pressure medication before PCI.

### PCI procedure and no-reflow definition

All STEMI patients received standardized treatment according to the Chinese standard version of the chest pain center [21], including administration of chewable 300 mg enteric-coated aspirin tablets and 180 mg ticagrelor/300mg clopidogrel. Two interventional cardiologists with over 5 years of emergency PCI experience conducted coronary angiography following the standard Judkins technique [22]. Based on the angiography results, coronary PCI was carried out in the culprit vessels. The specific procedure involved passing a coronary guidewire through the lesion, pre-dilating with a pre-dilation balloon, then implanting a coronary stent and releasing it at an appropriate pressure (atm). Finally, the decision to use a post-dilation balloon for further dilation was made as needed. After stent implantation, the two specialists evaluated the status of coronary blood flow (TIMI flow grade) using findings from coronary angiography. TIMI grades 0, 1, and 2 were defined as no-reflow, whereas TIMI grade 3 was defined as reflow [23]. In cases of disagreement between the two cardiologists, a discussion was held with a third cardiologists with over 10 years of emergency PCI experience. In cases of no reflow during PCI in STEMI patients, we treated no reflow by administering sodium nitroprusside through the punctured balloon in the coronary artery, and used the GP II/III inhibitor Tirofiban to treat no reflow in every patient experiencing it.

### Blood pressure measurement

Blood pressure measurements for all patients were collected at the time of admission or medical contact without any relevant drug intervention. This blood pressure data was recorded on the chest pain chart. Patients were classified into subgroups based on 20-mmHg increments for average BP values [24,25]: SBP <100 mmHg, 100 ≤SBP <120 mmHg, 120 ≤SBP <140 mmHg, 140 ≤ SBP <160 mmHg, and SBP ≥160 mmHg groups; or classified into DBP < 60 mmHg, 60≤ DBP <80 mmHg, 80 ≤DBP <100 mmHg, and DBP ≥ 100 mmHg.

### Statistical analysis

Multiple imputations were implemented using the “mice” package to address missing data. Continuous data were subjected to a normality test, with normally distributed quantitative data described as means ± standard deviation (SD), and non-normally distributed quantitative data presented as median and quartiles [M (Q_1_, Q_3_)]. Qualitative data were described using case numbers and percentages [n %]. One-way analysis of variance (ANOVA) or Kruskal-Wallis rank test was employed for comparing patient groups concerning continuous variable, while the chi-squared test was utilized for categorical variables based on the normal distribution of the data. Univariate logistic analysis was performed to assess the risk of no-reflow for each SBP or DBP subgroups. In the multivariable logistic regression analysis, we estimated the risk of no-reflow in SBP or DBP subgroups with adjustments for gender, age, smoking history, heart rate, the number of ST elevation leads greater than 3, hypertension, diabetes, Killip classification on admission, cardiac arrest occurred before PCI, heart failure during PCI, malignant arrhythmia during PCI, intravenous blood pressure medication before PCI, thrombus-shadow during coronary angiogram, lesion-vessel number, number-of-stents, and oral blood pressure medication before PCI [26,27,28,29]. The SBP or DBP subgroups with the lowest incidence of no-reflow were used as the reference group for the logistic regression model.

To minimize potential bias, we conducted three logistic regression models for sensitivity analyses to assess the robustness of the association between no-reflow and SBP or DBP subgroups. In Model 1, patients received oral and intravenous blood pressure medication before PCI were excluded, and sensitivity analysis was additionally adjusted for high-sensitivity cardiac troponin I (hscTnI). In Model 2, in addition to the variables in Model 1, preoperative conditions of transfer from another hospital, drug treatment by other hospital, time from onset of chest pain to the PCI procedure, and cardiac arrest that occurred before PCI and IABP were added. In Model 3, in addition to the variables in Model 2, oral and intravenous blood pressure medications were added. Statistical analyses were conducted using R, version 4.2.3 (R Foundation for Statistical Computing). A p-value of <0.05 was considered statistically significant.

## Results

### 1. Baseline characteristics

This study cohort included 1050 STEMI patients who underwent primary PCI at Xiangdong Hospital, Hunan Normal University between December 30, 2016, and May. 13, 2023. After excluding specific patients, 1025 patients were ultimately included in the study. The clinical and demographic characteristics of all patients are summarized in Table 1.

**Table 1:** Demographic and baseline characteristics of the patients by mean systolic blood pressure categories.


PARAMETER	TOTAL (n = 1025)	MEAN SYSTOLIC BP, MM HG					P-VALUE

		SBP <100 (n = 88)	100 ≤SBP <120 (n = 224)	120 ≤SBP <140 (n = 333)	140 ≤ SBP <160 (n = 217)	SBP ≥160 (n = 163)	

Men, n (%)	782 (76)	73 (83)	180 (80)	249 (75)	163 (75)	117 (72)	0.157

Transferred from other hospital, n (%)	334 (33)	35 (40)	76 (34)	118 (35)	65 (30)	40 (25)	0.062

Drug treatment by other hospital, n (%)	223 (22)	19 (22)	50 (22)	84 (25)	46 (21)	24 (15)	0.127

Age, Median (Q1,Q3)	64 (56, 71)	63.5 (58, 70)	64 (55.75, 70)	64 (55, 71)	66 (56, 72)	63 (54.5, 72)	0.683

HR, Median (Q1,Q3)), beats/min	75 (65, 87)	64.5 (47.75, 75.25)	70 (60, 82)	76 (66, 87)	80 (70, 93)	80 (68.5, 90)	<0.001

SBP, Median (Q1,Q3)	130 (114, 150)	91 (86.5, 95)	110 (105, 114)	130 (121, 132)	149 (144, 150)	170 (162.5, 180)	<0.001

DBP, Median (Q1,Q3)	80 (70, 90)	60 (50, 63)	70 (63.75, 76.25)	80 (70, 80)	90 (80, 97)	100 (90, 110)	<0.001

The number of ST elevation leads greater than 3, n (%)	582 (57)	60 (68)	135 (60)	182 (55)	118 (54)	87 (53)	0.105

hs-cTnI, Median (Q1,Q3)	0.51 (0.05, 4.14)	0.61 (0.06, 4.52)	0.34 (0.04, 5.64)	0.73 (0.06, 5.69)	0.69 (0.06, 3.77)	0.27 (0.06, 2.15)	0.115

Hypertension, n (%)	585 (57)	37 (42)	97 (43)	175 (53)	143 (66)	133 (82)	<0.001

Diabetes, n (%)	274 (27)	22 (25)	52 (23)	89 (27)	67 (31)	44 (27)	0.484

Smoking history, n (%)	367 (36)	33 (38)	83 (37)	114 (34)	85 (39)	52 (32)	0.597

Killip classification, n (%)							<0.001

1	766 (75)	45 (51)	169 (75)	266 (80)	168 (77)	118 (72)	

2	146 (14)	11 (12)	30 (13)	42 (13)	32 (15)	31 (19)	

3	43 (4)	3 (3)	9 (4)	13 (4)	10 (5)	8 (5)	

4	70 (7)	29 (33)	16 (7)	12 (4)	7 (3)	6 (4)	

Time from onset of chest pain to the PCI procedure, Median (Q1,Q3)	174 (83, 362)	143.5 (72.5, 229.75)	151.5 (79.75, 300)	193 (90, 439)	192 (95, 435)	156(75.5, 324.5)	0.003

Hb, Median (Q1,Q3)	137 (124.1, 147)	134.5(122.75,146.25)	134(123.75, 145.25)	136 (123, 146)	136 (125, 149)	141 (129.5, 152)	0.004

RBC, Median (Q1,Q3)	4.45 (4.07, 4.83)	4.42 (4.01, 4.76)	4.34 (4, 4.74)	4.38 (4.01, 4.78)	4.47 (4.1, 4.88)	4.62 (4.34, 5)	<0.001

MPV, Median (Q1,Q3)	10.6 (9.9, 11.3)	10.7 (9.7, 11.4)	10.6 (9.9, 11.12)	10.5 (9.8, 11.3)	10.6 (10, 11.3)	10.6 (10, 11.2)	0.682

WBC, Median (Q1,Q3)	10.3(8.22, 12.97)	11.91 (9.38, 15.36)	10.54 (8.54, 13.2)	10.19(7.92,12.55)	9.97 (8.12, 12.72)	9.98(7.98,12.53)	<0.001

NEUT, Median (Q1,Q3)	7.73(5.62, 10.48)	8.97 (6.7, 12.24)	8.07 (5.97, 10.57)	7.72(5.47, 10.36)	7.56 (5.56, 10.4)	6.96 (5.38, 9.78)	0.009

PLT, Median (Q1,Q3)	214 (177, 256)	213 (178.5, 273)	218 (178.75, 252)	210 (177, 256)	216 (177, 255)	213 (176, 257.5)	0.969

LYM, Median (Q1,Q3)	1.58 (1.12, 2.24)	1.73 (1.19, 2.67)	1.6 (1.12, 2.29)	1.55 (1.1, 2.11)	1.49 (1.06, 2.1)	1.67 (1.26, 2.59)	0.039

Glucose, Median (Q1,Q3)	7.22 (5.81, 9.88)	7.51 (5.91, 11.26)	7.33 (5.89, 9.83)	7.12 (5.77, 9.61)	7.1 (5.81, 9.11)	7.19(5.86,10.55)	0.671

Hyperlipemia, n (%)	125 (12)	7 (8)	21 (9)	37 (11)	37 (17)	23 (14)	0.065

Na, Median (Q1,Q3)	141.5 (139.1, 143.9)	142.2 (139.2, 144.5)	141.5 (139.3, 144.1)	141.2 (138.7, 143.8)	141.3 (138.9, 143.7)	142.1 (139.8, 143.8)	0.511

K, Median (Q1,Q3)	3.88 (3.62, 4.21)	3.92 (3.62, 4.26)	3.84 (3.55, 4.2)	3.86 (3.65, 4.21)	3.93 (3.67, 4.19)	3.9 (3.55, 4.22)	0.653

Cardiac arrest occurred before PCI, n (%)	30 (3)	7 (8)	7 (3)	9 (3)	5 (2)	2 (1)	0.081

Heart failure during PCI, n (%)	47 (5)	3 (3)	11 (5)	14 (4)	12 (6)	7 (4)	0.937

Malignant arrhythmia during PCI, n (%)	83 (8)	18 (20)	18 (8)	26 (8)	15 (7)	6 (4)	0.001

Intravenous blood pressure medication before PCI, n (%)	54 (5)	41 (47)	1 (0)	0 (0)	0 (0)	12 (7)	<0.001

Fibrinogen, Median (Q1,Q3)	3.12 (2.73, 3.64)	3.01 (2.67, 3.54)	3.08 (2.73, 3.5)	3.15 (2.69, 3.66)	3.2 (2.81, 3.71)	3.14 (2.71, 3.6)	0.203

Thrombus-shadow during coronary angiogram, n (%)	720 (70)	70 (80)	166 (74)	216 (65)	155 (71)	113 (69)	0.039

Lesion vessel number, n (%)							0.454

1	112 (11)	6 (7)	20 (9)	38 (11)	31 (14)	17 (10)	

2	197 (19)	21 (24)	45 (20)	58 (17)	45 (21)	28 (17)	

3	716 (70)	61 (69)	159 (71)	237 (71)	141 (65)	118 (72)	

Number of stents, n (%)							0.749

1	561 (55)	40 (45)	131 (58)	188 (56)	119 (55)	83 (51)	

2	344 (34)	33 (38)	72 (32)	107 (32)	72 (33)	60 (37)	

3	90 (9)	9 (10)	15 (7)	30 (9)	19 (9)	17 (10)	

4	24 (2)	5 (6)	5 (2)	6 (2)	6 (3)	2 (1)	

5	6 (1)	1 (1)	1 (0)	2 (1)	1 (0)	1 (1)	

IABP, n (%)	48 (5)	10 (11)	9 (4)	13 (4)	8 (4)	8 (5)	0.041

Oral blood pressure medication before PCI, n (%)	164 (16)	10 (11)	35 (16)	54 (16)	30 (14)	35 (21)	0.212


Abbreviations: CI, confidence interval; HR, heart rate; DBP, diastolic blood pressure; SBP, systolic blood pressure; hs-cTnI, high-sensitivity cardiactroponinI; Hb, Hemoglobin; RBC, red blood cell count; MPV, Mean platelet volume; WBC, white blood cell count; NEUT, neutrophil count; PLT, platelet count; LYM, lymphocyte count; PCI, percutaneous coronary intervention; IABP, intra-aortic ballon pump.

The patients were categorized into SBP groups and DBP groups based on blood pressure recorded at admission or medical contact without drug intervention. Patients with low SBP (≤120 mmHg) used more intravenous blood pressure medication before PCI and IABP during PCI, and had a higher peripheral white blood count, neutrophil count, lymphocyte count, Killip classification, and rate of malignant arrhythmia during PCI (all p < 0.05). Meanwhile, they had a lower heart rate, hemoglobin, red blood count and the rate of comorbidities of hypertension (all p < 0.05) (Table 1). However, patients with low DBP (≤80 mmHg) constituted a cohort that tended to be older and transferred from other hospitals, with a higher cardiac Troponin I level compared to patients with high DBP (all p < 0.05) (supplement table1).

### 2. Association between systolic blood pressure and no-reflow

The incidence of no-reflow were 40.9%, 22.3%, 17.1%, 24.9% and 27.6% in the groups with SBP <100 mmHg, 100 ≤SBP <120 mmHg, 120 ≤SBP <140 mmHg, 140 ≤ SBP <160 mmHg, and SBP ≥160 mmHg (Table 2). Since the group with 120 ≤SBP <140 mmHg had the lowest no-reflow incidence, it was chosen as the reference groups for further analysis.

**Table 2:** Crude and adjusted odds ratios for mean systolic blood pressure and diastolic blood pressure categories.


CATEGORIES	BP QUARTILES (MMHG)	NO REFLOW (N/ TOTAL)	INCIDENCE	ODDS RATIO(CI95%)			

				CRUDE	P-VALUE	ADJUSTED	P-VALUE

Systolic blood pressure	SBP <100	36/88	0.409	3.35 (2.00,5.59)	<0.001	3.64 (1.84,7.21)	<0.001

	100 ≤SBP <120	50/224	0.223	1.39 (0.91,2.13)	0.127	1.40 (0.89,2.20)	0.145

	120 ≤SBP <140	57/333	0.171	ref	–	ref	–

	140 ≤ SBP <160	54/217	0.249	1.60 (1.05,2.44)	0.027	1.62 (1.03,2.54)	0.036

	SBP ≥160	45/163	0.276	1.85 (1.18,2.88)	0.007	2.07 (1.27,3.36)	0.003

Diastolic blood pressure	DBP <60	24/67	0.358	3.17 (1.78,5.56)	<0.001	3.28 (1.63,6.49)	<0.001

	60≤ DBP <80	105/408	0.257	1.97 (1.39,2.80)	<0.001	1.87 (1.28,2.74)	0.001

	80 ≤DBP <100	61/407	0.150	ref	–	ref	–

	DBP ≥ 100	52/143	0.364	3.24 (2.09,5.02)	<0.001	3.36 (2.07,5.46)	<0.001


Abbreviations: CI, confidence interval; OR, odds ratio; DBP, diastolic blood pressure; SBP, systolic blood pressure. Models adjusted for gender, age, smoking history, heart rate, the number of ST elevation leads greater than 3, hypertension, diabetes, Killip classification on admission, cardiac arrest occurred before PCI, heart failure during PCI, malignant arrhythmia during PCI, intravenous blood pressure medication before PCI, Thrombus-shadow during coronary angiogram, Lesion-vessel number, number of stents, oral blood pressure medication before PCI.

To explore the association between SBP levels and no-reflow, the logistic regression model was conducted in SBP groups. Compared to patients with 120 ≤SBP <140 mmHg, those in the groups with SBP <100 mmHg, 100 ≤SBP <120 mmHg, 140 ≤ SBP <160 mmHg, and SBP ≥160 mmHg had an increased incidence of no-reflow, with ORs of 3.35 (95% CI 2.00–5.59), 1.39(95% CI 0.91–2.13), 1.60(95% CI 1.05–2.44), and 1.85(95% CI 1.85–2.88), respectively (Table 2).

**Figure 1:**
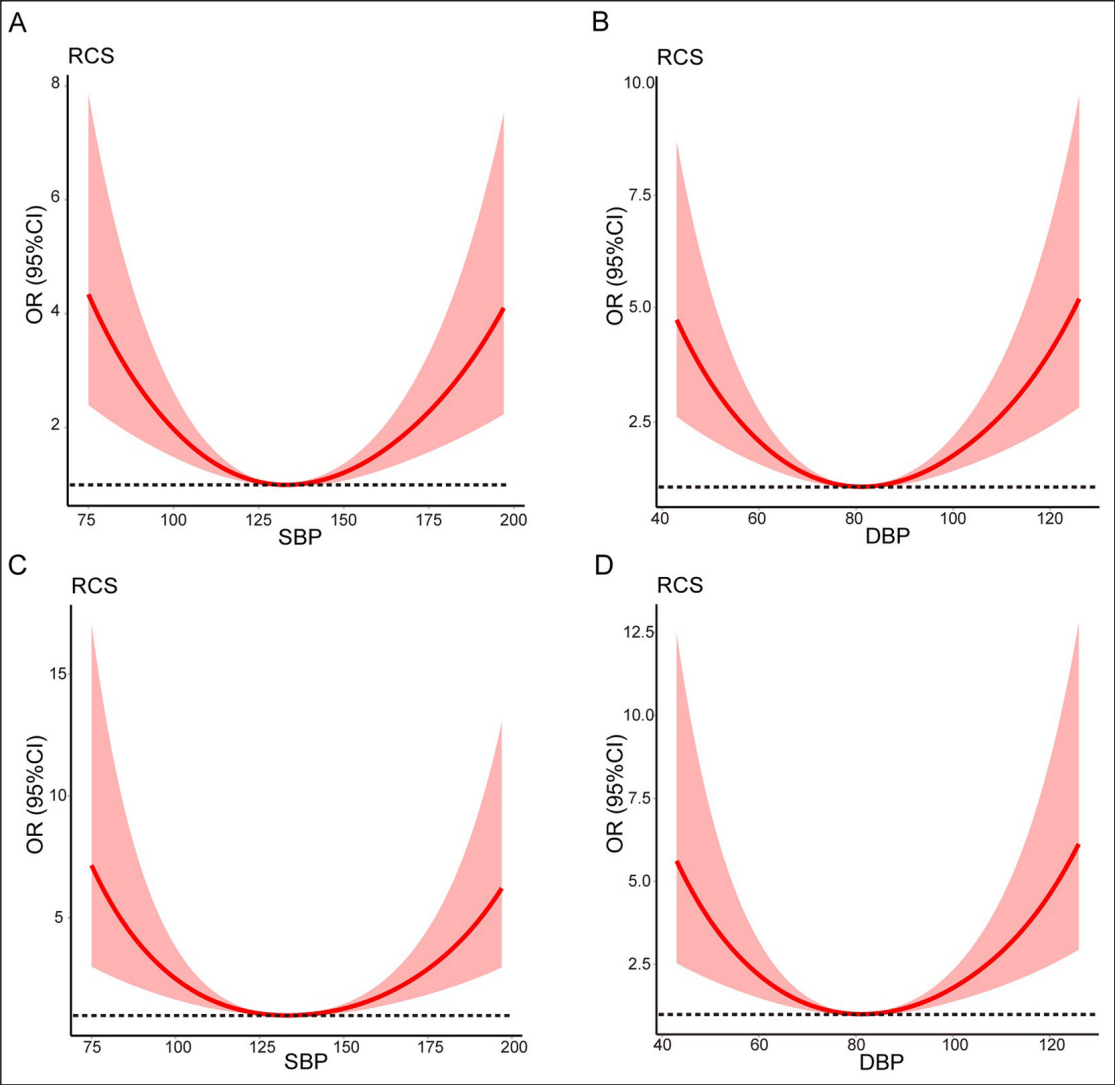
The associations of blood pressure with no-reflow. A: The association between systolic blood pressure (SBP) and no-reflow; B: The association between diastolic blood pressure (DBP) and no-reflow; C: The association between systolic blood pressure (SBP) and no-reflow after adjusted; D: The association between diastolic blood pressure (DBP) and no-reflow after adjusted; Adjusted odds ratio was calculated based on the logsitic model with adjustment of gender, age, smoking history, heart rate, a number of ST elevation leads greater than 3, hypertension, diabetes, Killip classification on admission, Cardiac arrest occurred before PCI, heart failure during PCI, malignant arrhythmia during PCI, intravenous blood pressure medication before PCI, Thrombus-shadow during coronary angiogram, Lesion-vessel number, Number of stents, oral blood pressure, and medication before PCI. The 95% confidence intervals were represented by shaded regions. Abbreviations: CI, confidence interval; SBP, systolic blood pressure; DBP, diastolic blood pressure; OR, odds ratio. PCI, percutaneous transluminal coronary intervention.

After adjusting for gender, age, smoking history, heart rate, hypertension, diabetes, Killip classification, the number of ST elevation leads greater than 3, heart arrest, heart failure during PCI, malignant arrhythmia during PCI, intravenous blood pressure medication before PCI, thrombus-shadow during coronary angiogram, lesion vessel number, and number of stents and oral blood pressure medication before PCI as confounders, the associations of no-reflow incidence with SBP groups were consistent. In summary, patients with SBP <100 mmHg and SBP ≥140 mmHg were associated with significantly higher incidence of no-reflow. Cubic splines analysis for the relationship between SBP and outcomes showed a U-shaped shaped curve, indicating an increased incidence of no-reflow at low and high SBP (Figure 1).

### 3. The association between diastolic blood pressure and no-reflow

The incidences of no-reflow were 35.8%, 25.7%, 15.0%, and 36.4% in the groups with DBP < 60 mmHg, 60 ≤ DBP < 80 mmHg, 80 ≤ DBP <100 mmHg, and DBP≥100 mmHg (Table 2). Since the group with DBP 80 ≤ DBP <100 mmHg had the lowest no-reflow incidence, it was chosen as reference groups for further analysis.

The logistic regression model was conducted in DBP groups to explore the association between DBP levels and incidence of no-reflow. Compared to patients with 80 ≤ DBP <100 mmHg, those in the groups with DBP <60 mmHg, 60 ≤ DBP <80 mmHg, and DBP ≥100 mmHg had an increased incidence of no-reflow, with ORs 3.17 (95% CI 1.78–5.56), 1.97 (95% CI 1.39–2.80), and 3.24 (95% CI 2.09–5.02), respectively (Table 2). After adjusted gender, age, smoking history, heart rate, hypertension, diabetes, Killip classification, the number of ST elevation leads greater than 3, heart arrest, heart failure during PCI, malignant arrhythmia during PCI, intravenous blood pressure medication before PCI, thrombus-shadow during coronary angiogram, lesion vessel number, and number of stents and oral blood pressure medication before PCI as confounders, the associations of the no-reflow incidence with DBP groups were consistent. In summary, patients with DBP <80 mmHg and with DBP ≥100 mmHg were associated with a significantly higher incidence of no-reflow. The cubic splines analysis for the relationship between DBP and outcomes showed a U-shaped shaped curve, with an increased incidence of no-reflow at low and high DBP (Figure 1).

### 4. Sensitivity analysis

We conducted three sensitivity analyses to further confirm the robustness of the association between blood pressure before treatment and no-reflow of STEMI. In Model 1, after additionally adjusting for gender, age, smoking history, heart rate, hs-cTnI, hypertension, diabetes, Killip classification, the number of ST elevation leads greater than 3, heart arrest, heart failure during PCI, malignant arrhythmia during PCI, thrombus-shadow during coronary angiogram, lesion vessel number, and number of stents, patients with SBP <100 mmHg, 140 ≤ SBP <160 mmHg and SBP ≥160 mmHg exhibited an increased incidence of no-reflow, with ORs 3.60 (95% CI 1.99–5.33), 1.69 (95% CI 1.07–2.65), and 2.26 (95% CI 1.39–3.70) in comparison to patients with 120 ≤ SBP < 140 mmHg, respectively (Table 3). In the DBP subgroups, compared to patients with 80 ≤ DBP <100 mmHg, those with DBP <60 mmHg, 60≤ DBP <80 mmHg, and DBP ≥ 100 mmHg showed an increased incidence of no-reflow, with ORs 3.58 (95% CI 1.87–6.79), 1.83 (95% CI 1.26–2.69), and 3.69 (95% CI 2.28–6.01), respectively (Table 3). The associations of no-reflow with SBP and DBP subgroups remained consistent and statistically significant in Model 2 and Model 3 (Table 3).

**Table 3:** Adjusted odds ratios for mean systolic and diastolic blood pressure categories in sensitivity analysis.


CATEGORIES	BP QUARTILES (MMHG)	ODDS RATIO (CI95%)					

		MODEL1	P-VALUE	MODEL2	P-VALUE	MODEL3	P-VALUE

Systolic blood pressure	SBP <100	3.60 (1.99, 6.53)	<0.001	3.79 (2.07, 6.97)	<0.001	3.81 (1.89, 7.70)	<0.001

	100 ≤SBP <120	1.40 (0.89, 2.21)	0.145	1.49 (0.93, 2.36)	0.094	1.49 (0.93, 2.36)	0.093

	120 ≤SBP <140	ref	–	ref	–	ref	–

	140 ≤ SBP <160	1.69 (1.07, 2.65)	0.023	1.75 (1.11, 2.78)	0.017	1.78 (1.12, 2.82)	0.015

	SBP ≥160	2.26 (1.39, 3.70)	0.001	2.37 (1.44, 3.90)	<0.001	2.38 (1.44, 3.94)	<0.001

Diastolic blood pressure	DBP <60	3.58 (1.87, 6.79)	<0.001	4.05 (2.09, 7.75)	<0.001	3.77 (1.85, 7.58)	<0.001

	60≤ DBP <80	1.83 (1.26, 2.69)	0.002	1.84 (1.26, 2.72)	0.002	1.83 (1.25, 2.71)	0.002

	80 ≤DBP <100	ref	–	ref	–	ref	–

	DBP ≥ 100	3.69 (2.28, 6.01)	<0.001	3.95 (2.41, 6.50)	<0.001	3.89 (2.37, 6.41)	<0.001


Abbreviations: CI, confidence interval; DBP, diastolic blood pressure; oR, odds ratio; SBP, systolic blood pressure. Model 1 adjusted for gender, age, smoking history, heart rate, the number of ST elevation leads greater than 3, hscTnI, hypertension, diabetes, Killip classification on admission, cardiac arrest occurred before PC, heart failure during PCI, malignant arrhythmia during PCI, Thrombus-shadow during coronary angiogram, Lesion-vessel number, number of stents. Model 2 adjusted for gender, age, smoking history, heart rate, number of ST elevation leads greater than 3, transferred from other hospital, drug treatment by other hospital, hscTnI, hypertension, diabetes, Killip classification on admission, time from onset of chest pain to the PCI procedure, cardiac arrest occurred before PCI, heart failure during PCI, malignant arrhythmia during PCI, Thrombus-shadow during coronary angiogram, Lesion-vessel number, number of stents, and IABP. Model 3 adjusted for gender, age, smoking history, heart rate, number of ST elevation leads greater than 3, transferred from other hospital, Drug treatment by other hospital, hscTnI, hypertension, diabetes, Killip classification on admission, time from onset of chest pain to the PCI procedure, cardiac arrest occurred before PCI, heart failure during PCI, malignant arrhythmia during PCI, intravenous blood pressure medication before PCI, Thrombus-shadow during coronary angiogram, Lesion-vessel number, number of stents, IABP, and oral blood pressure medication before PCI.

## Discussion

This study represents the first exploration of the association between pre-PCI blood pressure and intraoperative no-reflow during PCI. We conducted a retrospective study of 1025 patients undergoing emergency PCI at the chest pain center of in Xiangdong Hospital, Hunan Normal University, China. After adjusting for potential confounders using a multivariate logistic regression model, we found that lowest no-reflow rates were in the 120 ≤ SBP < 140 mmHg and 80 ≤ DBP <100 mmHg groups. These findings offer valuable insights into the optimal range of blood pressure control before PCI.

Previous research suggests that microvascular thrombus formation, spasm, neutrophil blockage, and/or tissue edema are proven causes of coronary no-reflow, with microvascular thrombus formation considered a key factor [30]. When performing PCI on ischemic vessels under severe thrombus load, no-reflow often occurs, closely associated with microvascular thrombus [31,32]. Fragments of thrombi crushed post-PCI may shift distally, or ischemia-reperfusion-induced intravascular microthrombus formation can result in coronary no-reflow [33,34]. Excessive reperfusion pressure induced by high blood pressure may exacerbate microembolism of the microvasculature in the microvascular system by pushing aggregates of red blood cells, neutrophils, and platelets distally into smaller diameter vessels, intensifying no-reflow [18,20]. Conversely, low perfusion pressure can lead to decreased coronary microvascular blood flow, combined with reperfusion injury, increasing the risk of microthrombus formation and subsequent no-reflow [35]. Blood pressure, by affecting coronary perfusion pressure, plays a crucial role in the occurrence of coronary no-reflow. For operators performing emergency PCI for STEMI, close attention to blood pressure management is essential, allowing prompt adjustments without causing delays in the procedure [36]. Previous research by John N Nanas et al. demonstrated that reducing mean arterial pressure during reperfusion decreased coronary blood flow and increased myocardial infarction extent in pigs [37]. This is primarily due to insufficient arterial pressure to overcome the high resistance of microvasculature within the no-reflow zone. Surprisingly, their subsequent study revealed that increasing perfusion pressure did not alleviate the no-reflow phenomenon compared to normal perfusion pressure; instead, it exacerbated no-reflow and increased infarct size [20]. Our study further identified a U-shaped relationship between admission blood pressure and no-reflow incidence among patients undergoing primary PCI for STEMI. Both excessively low and high systolic and diastolic blood pressure were associated with an increased occurrence of no-reflow. Additionally, our study found that changes in DBP had a stronger correlation with no-reflow when DBP was below 80 mmHg or above 100 mmHg (P < 0.01). Previous studies have also indicated that coronary perfusion predominantly occurs during diastole, making low DBP more likely to result in decreased coronary blood flow and increased risk of no-reflow [38,39]. Moreover, our study population comprised elderly patients with an average age of 64 years, wherein arterial wave reflection and arterial stiffness were increased. Changes in DBP further affected coronary perfusion and thus influence no-reflow [40].

Previous studies have shown that optimal blood pressure control in patients with AMI to be critical for their prognosis [41]. The association between SBP or DBP and adverse outcomes followed a U-shaped or J-shaped curve [27,42,43,44]. Both low blood pressure and hypertension can increase the incidence of adverse events independently of other influencing factors [29,45]. For AMI patients after emergency PCI, the occurrence of no-reflow is closely related to the aforementioned adverse reactions [6,7,8]. Our study is the first to investigate the relationship between blood pressure and no-reflow. Interestingly, we found that the relationship between SBP or DBP and no-reflow also followed a U-shaped association. Furthermore, data analysis from the ONTARGET and TRANSCEND trials in patients at high cardiovascular risk have shown that an SBP between 120 mmHg and 140 mmHg had the lowest incidence of a cardiovascular event [46]. Therefore, our study further expands and refines the evidence on optimal blood pressure control in AMI patients.

With the proliferation of chest pain centers (CPCs), effectively preventing and reducing coronary no-reflow in patients with STEMI after timely and effective PCI treatment has become an urgent problem to solve [47]. Since the key to vascular recanalization in patients with AMI is to solve the problem of coronary blood flow, the mere implantation of a stent will not achieve the goal of timely reperfusion in the CPC. Therefore, the latest opinion has incorporated door-to-flow time into the implementation criteria of CPC. In contrast to other articles, the population of our study is STEMI patients who underwent emergency PCI according to the national standard version of the CPC procedure, ensuring uniform implementation standards and reducing the bias caused by treatment and intervention measures. This approach provides valuable insights for optimizing CPCs. In adherence to the CPC principle that time is of the essence for heart muscle preservation, we limited the scope of screened variables to clinical data quickly obtainable before and during the operation, facilitating a swift judgment of the optimal blood pressure. This makes our study more akin to real-world research.

## Limitations

Several limitations need consideration in interpreting our findings. Firstly, causality cannot be assumed or confirmed in our retrospective analysis. Secondly, despite extensive adjustments, potentially unmeasured variables, such as social status, job stress, and mental health, were not fully accounted for, contributing to residual confounding bias. Thirdly, this study included only one pre-PCI blood pressure measurement, and detailed intraoperative blood pressure changes were not recorded. Future studies will incorporate multiple time-point blood pressure measurements. Fourth, our cohort originated from a single center, potentially limiting the generalizability of our results to other regions. Fifth, despite multiple sensitivity analyses were conducted, the imputation of missing data to explore the association between blood pressure at admission and no-reflow has been limited, introducing potential bias, and further restricting generalizability to other populations [48]. Larger, more comprehensive studies of patients with STEMI are necessary. Sixth, the small sample size may diminish the strength and reliability of our hypothesis validation [49]. Future studies with larger populations are required to confirm the association between pre-PCI blood pressure and no-reflow.

## Conclusion

Our study identified a U-shaped association between BP before the PCI operation and no-reflow in STEMI patients. Patients with a mean SBP ranging from 120 mmHg to 140 mmHg and a DBP from 80 mmHg to 100 mmHg before the PCI demonstrated the lowest no-reflow. This study provides targets for blood pressure control before PCI to STEMI patients to mitigate no-reflow.

## Additional Files

The additional files for this article can be found as follows:

10.5334/gh.1309.s1 Supplementary Figure 1. The screen process of participants in this study.

10.5334/gh.1309.s2 Supplementary Table 1. Demographic and baseline characteristics of the patients by mean diastolic blood pressure categories.
