# Correction: Association of Pre-PCI Blood Pressure and No-Reflow in Patients with Acute ST-Elevation Coronary Infarction

**DOI:** 10.5334/gh.1583

**Published:** 2026-09-09

**Authors:** Xiaobo Li, Chen Yu, Li Lei, Xuewei Liu, Yejia Chen, Yutian Wang, ShiFeng Qiu, Jiancheng Xiu

**Affiliations:** 1 Department of Cardiology, Nanfang Hospital, Southern Medical University, Guangzhou, Guangdong, China; 2 Department of Cardiology, Xiangdong Hospital, Hunan Normal University, Liling, Hunan, China; 3 The Tenth Affiliated Hospital of Southern Medical University (Dongguan People’s Hospital), Southern Medical University, Dongguan, Guangdong, China

**Keywords:** STEMI (ST-elevation acute coronary infarction), no-reflow, SBP(Systolic blood pressure), DBP(Diastolic blood pressure)

## Abstract

This article details a correction to: Li X, Yu C, Lei L, Liu X, Chen Y, Wang Y, Qiu S, Xiu J. Association of Pre-PCI Blood Pressure and No-Reflow in Patients with Acute ST-Elevation Coronary Infarction. *Global Heart*. 2024;19(1):28. DOI: https://doi.org/10.5334/gh.1309 (1).

## Correction

The statistical analysis of this study was performed via binary logistic regression, and all effect sizes were reported as odds ratios (OR). However, an incorrect statistical term was mistakenly written in the manuscript proofreading stage.

Page 1, Line 2 of the Abstract’s Results section:

Original text: adjusted hazard ratio (OR)

Corrected text: adjusted odds ratio (OR)

Notably, all OR values, 95% confidence intervals, P values, U-shaped association trend and core clinical conclusions of this research remain unchanged. This wording error does not alter any interpretation of our findings. All co-authors have reviewed and approved this correction. We sincerely apologize for the confusion brought to readers.

## References

[1] Li X, Yu C, Lei L, Liu X, Chen Y, Wang Y, Qiu S, Xiu J. Association of Pre-PCI Blood Pressure and No-Reflow in Patients with Acute ST-Elevation Coronary Infarction. Global Heart, 2024. 19(1):28. DOI: 10.5334/gh.1309 38464557 PMC10921965

