# Supplementary figures and images for: Association of Pre-PCI Blood Pressure and No-Reflow in Patients with Acute ST-Elevation Coronary Infarction
Source: Glob Heart. 2024 Mar 4;19(1):28. doi: 10.5334/gh.1309 (PMC10921965; doi:10.5334/gh.1309)

supplement figure 1 The screen process of participants in this study.

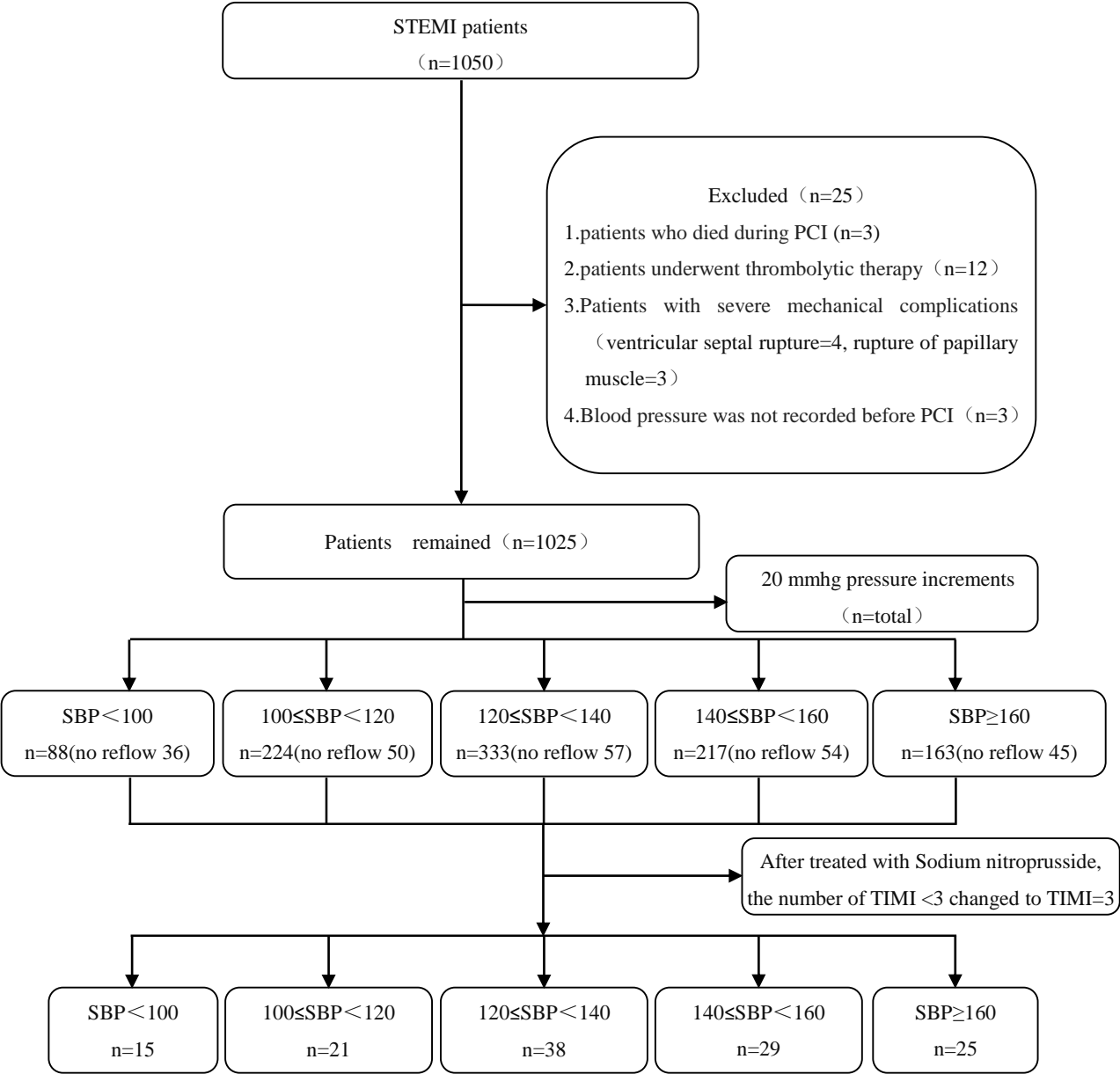

Supplement: Supplementary Figure 1. — The screen process of participants in this study. [file gh-19-1-1309-s1.pdf]
