# Supplementary material for: Association of Pre-PCI Blood Pressure and No-Reflow in Patients with Acute ST-Elevation Coronary Infarction
Source: Glob Heart. 2024 Mar 4;19(1):28. doi: 10.5334/gh.1309 (PMC10921965; doi:10.5334/gh.1309)
Supplement: Supplementary Table 1. — Demographic and baseline characteristics of the patients by mean diastolic blood pressure categories. [file gh-19-1-1309-s2.pdf]

supplement table1 :Demographic and baseline characteristics of the patients by mean diastolic blood pressure categories.

Variables	Total (n = 1025)	Mean diastolic blood pressure, mmHg				p-value
		DBP < 60 (n = 67)	60≤ DBP <80 (n = 408)	80 ≤DBP <100(n = 407)	DBP ≥ 100 (n = 143)	
Men,n (%)	782 (76)	55 (82)	311 (76)	304 (75)	112 (78)	0.542
Transfered from other hospital, n (%)	334 (33)	24 (36)	160 (39)	119 (29)	31 (22)	< 0.001
Drug treatment by other hospital, n (%)	223 (22)	17 (25)	108 (26)	78 (19)	20 (14)	0.006
Age, Median (Q1,Q3)	64 (56, 71)	65 (59, 72)	65 (56, 70.25)	65 (56, 72)	58 (50, 68)	< 0.001
HR, median (Q1,Q3)), beats/min	75 (65, 87)	65 (50, 75)	71.5 (61, 84)	78 (69, 88.5)	85 (71, 99.5)	< 0.001
SBP, Median (Q1,Q3)	130 (114, 150)	93 (82, 101)	118 (107, 128)	140 (130, 150)	165 (151, 180)	< 0.001
DBP, Median (Q1,Q3)	80 (70, 90)	52 (46, 55.5)	70 (67, 75)	85 (80, 90)	108 (101, 112)	< 0.001
The number of ST elevation leads greater than 3, n (%)	582 (57)	42 (63)	246 (60)	209 (51)	85 (59)	0.04
Hs-cTnI, Median (Q1,Q3)	0.51 (0.05, 4.14)	0.16 (0.04, 2.72)	0.81 (0.05, 7.47)	0.57 (0.05, 3.77)	0.26 (0.06, 1.9)	0.036
Hypertension, n (%)	585 (57)	34 (51)	198 (49)	244 (60)	109 (76)	< 0.001
Diabete, n (%)	274 (27)	13 (19)	110 (27)	105 (26)	46 (32)	0.242
Smoke, n (%)	367 (36)	20 (30)	146 (36)	143 (35)	58 (41)	0.472
Killip classification, n (%)						< 0.001
1	766 (75)	37 (55)	306 (75)	320 (79)	103 (72)	
2	146 (14)	8 (12)	55 (13)	58 (14)	25 (17)	
3	43 (4)	2 (3)	15 (4)	16 (4)	10 (7)	
4	70 (7)	20 (30)	32 (8)	13 (3)	5 (3)	
Time from onset of chest pain to the PCI procedure, Median (Q1,Q3)	174 (83, 362)	119 (59, 224)	181 (88, 379)	180 (87.5, 412.5)	145 (73, 312.5)	0.005
Hb, Median (Q1,Q3)	137 (124.1, 147)	133 (122, 145.5)	133 (121, 144)	137 (125, 148)	144 (136, 156.5)	< 0.001
RBC, Median (Q1,Q3)	4.45 (4.07, 4.83)	4.3 (3.99, 4.67)	4.3 (3.95, 4.7)	4.46 (4.12, 4.84)	4.79 (4.5, 5.2)	< 0.001
MPV, Median (Q1,Q3)	10.6 (9.9, 11.3)	10.6 (9.8, 11.25)	10.5 (9.8, 11.2)	10.6 (9.9, 11.4)	10.6 (10.05, 11.1)	0.299
WBC, Median (Q1,Q3)	10.3 (8.22, 12.97)	11.21 (9.47, 13.87)	10.54 (8.42, 13.23)	9.67 (7.78, 12.3)	10.83 (8.66, 13.28)	< 0.001
NEUT, Median (Q1,Q3)	7.73 (5.62, 10.48)	8.13 (6.44, 11.55)	8.06 (6.04, 10.62)	7.24 (5.21, 9.76)	7.92 (5.83, 10.73)	0.001
PLT, Median (Q1,Q3)	214 (177, 256)	216 (179.5, 276)	213 (179, 252)	213 (173, 256)	215 (189.5, 257)	0.665
LYM, Median (Q1,Q3)	1.58 (1.12, 2.24)	1.64 (1.29, 2.66)	1.56 (1.07, 2.15)	1.57 (1.09, 2.22)	1.68 (1.26, 2.52)	0.024

Glucose, Median (Q1,Q3)	7.22 (5.81, 9.88)	7.13 (5.85, 9.78)	7.31 (5.74, 9.57)	7.19 (5.81, 9.84)	7.35 (6, 11.03)	0.614
Hyperlipemia, n (%)	125 (12)	5 (7)	44 (11)	54 (13)	22 (15)	0.266
Na, Mean ± SD	141.5 (139.1, 143.9)	141.6 (139.0, 144.5)	141.8 (139.4, 144.2)	141.2 (138.9, 143.8)	142.1 (138.8, 143.4)	0.321
K, Median (Q1,Q3)	3.88 (3.62, 4.21)	3.91 (3.57, 4.28)	3.85 (3.62, 4.18)	3.89 (3.64, 4.21)	3.92 (3.67, 4.24)	0.477
Heart arrest, n (%)	30 (3)	3 (4)	17 (4)	7 (2)	3 (2)	0.125
Heart failure during PCI, n (%)	47 (5)	1 (1)	19 (5)	19 (5)	8 (6)	0.654
Malignant arrhythmia during PCI, n (%)	83 (8)	9 (13)	41 (10)	25 (6)	8 (6)	0.047
Intravenous blood pressure medication before PCI, n (%)	54 (5)	27 (40)	15 (4)	1 (0)	11 (8)	< 0.001
Fibrinogen, Median (Q1,Q3)	3.12 (2.73, 3.64)	2.91 (2.58, 3.5)	3.12 (2.74, 3.63)	3.15 (2.76, 3.68)	3.14 (2.68, 3.62)	0.140
Thrombus-shadow during coronary angiogram, n (%)	720 (70)	51 (76)	288 (71)	283 (70)	98 (69)	0.699
Lesion vessel number, n (%)						0.110
1	112 (11)	3 (4)	45 (11)	42 (10)	22 (15)	
2	197 (19)	16 (24)	76 (19)	71 (17)	34 (24)	
3	716 (70)	48 (72)	287 (70)	294 (72)	87 (61)	
Number of stents, n (%)						0.102
1	561 (55)	33 (49)	231 (57)	210 (52)	87 (61)	
2	344 (34)	21 (31)	135 (33)	145 (36)	43 (30)	
3	90 (9)	12 (18)	26 (6)	43 (11)	9 (6)	
4	24 (2)	1 (1)	12 (3)	8 (2)	3 (2)	
5	6 (1)	0 (0)	4 (1)	1 (0)	1 (1)	
IABP, n (%)	48 (5)	5 (7)	19 (5)	18 (4)	6 (4)	0.730
Oral blood pressure medication before PCI, n (%)	164 (16)	13 (19)	59 (14)	64 (16)	28 (20)	0.443

Abbreviations: CI, confidence interval; HR, heart rate; DBP, diastolic blood pressure; SBP, systolic blood pressure; hs-cTnI, high-sensitivitycardiactroponinI; Hb,Hemoglobin; RBC, red blood cell count; MPV, Mean platelet volume; WBC, white blood cell count; NEUT, neutrophil count; PLT, platelet count; LYM, lymphocyte count; IABP, intra-aortic ballon pump.
